# Comparison of cardiovascular magnetic resonance feature tracking and tagging for the assessment of left ventricular systolic strain in acute myocardial infarction

**DOI:** 10.1186/1532-429X-17-S1-P102

**Published:** 2015-02-03

**Authors:** Jamal N Khan, Anvesha Singh, Sheraz A Nazir, Prathap Kanagala, John P Greenwood, Anthony Gershlick, Gerry P McCann

**Affiliations:** Cardiovascular Sciences, University of Leicester, Leicester, UK; University of Leeds, Leeds, UK

## Background

Contractile dysfunction following ST-segment elevation myocardial infarction (STEMI) predicts prognosis. CMR-measured tagging is the gold standard technique for myocardial strain assessment. Strain offers greater accuracy in detecting dysfunctional myocardium than ejection fraction and visual measures of regional function. Tagging however requires acquisition of additional sequences and time-consuming post-processing. FT tracks features of interest along contour lines on routinely acquired steady-state free-precession (SSFP) cine images. There are no published strain data using FT in acute STEMI. This study aimed to assess the feasibility of FT measured global and segmental circumferential (Ecc) and longitudinal (Ell) strain assessment post acute STEMI and compare values to those obtained with tagging.

## Methods

CMR at 1.5T was performed in 24 acute STEMI patients. SSFP, T2wSTIR (oedema) and Late Gadolinium Enhancement (infarct) imaging were performed in long-axis views and contiguous short-axis slices covering the left ventricle. Pre-contrast short and long-axis tagged images were acquired using a spatial modulation of magnetization gradient-echo sequence. Segmental infarct and oedema were calculated as a percentage of segmental area. Global and segmental Ecc and Ell were assessed using FT (*Tomtec Image Arena*, *Germany*) and tagging (*InTag*, *France*). Ecc and Ell on FT and tagging were correlated with total and segmental infarct size and oedema, assessed in relation to segmental transmural infarction, and compared in infarct, adjacent and remote segments. Intraobserver and interobserver variability of the techniques were compared.

## Results

**Figure 1:**
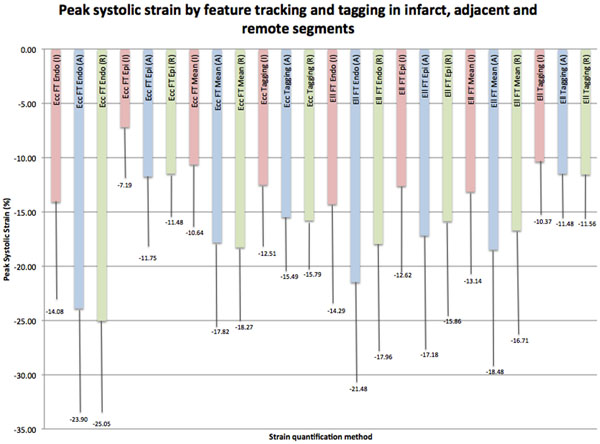
**Peak systolic strain by feature tracking and tagging in infarct, adjacent and remote segments.** Endo = endocardial FT contour derived, Epi = epicardial FT contour derived, Mean = mean of strain obtained when both endocardial and epicardial FT contours drawn

All segments tracked satisfactorily with FT (p<0.001). Analysis time per patient was shorter with FT (Table 1). Global Ecc and Ell were higher with FT than with tagging, apart from FT Ecc using the average of endocardial and epicardial contours. Intraobserver and interobserver agreement for global strain (ICCs>0.90) were excellent for FT but interobserver agreement for tagging was lower (ICC>0.73). Interobserver and intraobserver agreement for segmental strain were good for both techniques (ICC >0.7) apart from tagging Ell, which was poor (ICC=0.15). FT-derived Ecc significantly correlated with total infarct size (r=0.44, p=0.03) and segmental infarct extent (r=0.44, p<0.01). Segmental oedema correlated significantly with FT-derived strain, and stronger than with tagging-derived strain. FT-derived strain more accurately predicted transmural infarction compared with tagging-derived strain, on ROC analysis. Strain in infarcted segments was significantly lower than in adjacent segments using all methods except tagging-derived Ell (Figure 1).

**Table 1:** Myocardial tracking and quantification time by method

	Feature Tracking (FT)	Tagging	p value (FT v Tagging)
Short-axis cine segments excluded	0 (0%)	18 (5%)	<0.001
Long-axis cine segments excluded	0 (0%)	81 (21%)	<0.001
Number of excluded short-axis segments in infarct core	0 (0%)	3/18 (17%)	<0.001
Number of excluded long-axis segments in infarct core	0 (0%)	14/81 (17%)	<0.001
Analysis time per patient (minutes)	25.3±3.7	33.4±5.6	<0.001
Post-processing time per patient (minutes)	12.9±2.0	30.3±5.9	<0.001
Total analysis time (minutes)	38.2±3.8	63.7±10.3	<0.001

Infarction defined where ≥1% segmental late gadolinium enhancement.

## Conclusions

FT-derived global Ecc and Ell measurement in acute STEMI is feasible and robust. FT-derived strain is quicker to analyse, tracks myocardium better, has better interobserver variability and stronger correlations with infarct and oedema than tagging and more accurately predicts transmural infarction.

## Funding

National Institute for Health Research (NIHR).

